# Supplementary figures and images for: Quantifying β-catenin subcellular dynamics and cyclin D1 mRNA transcription during Wnt signaling in single living cells
Source: eLife. 2016 Nov 23;5:e16748. doi: 10.7554/eLife.16748 (PMC5161448; doi:10.7554/eLife.16748)

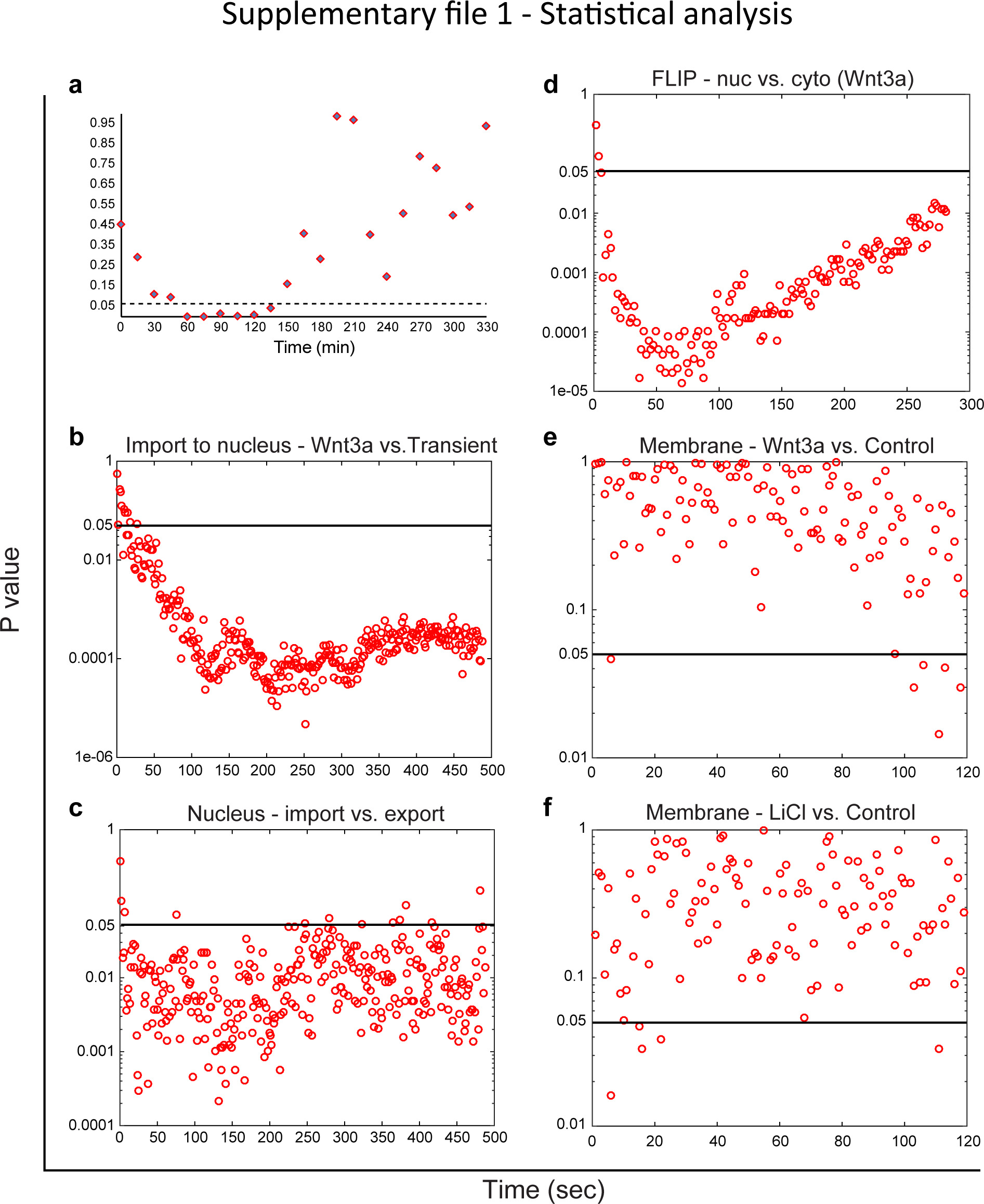

Supplement: Supplementary file 1. — (a) The statistical significance p values (t test) at each time point for the percentage of cells showing an active CCND1-MS2 gene (refers to Figure 1b) between control and Wnt3a-treated cells. (b-f) Mann-Whitney test for comparison between two independent FRAP/FLIP experiments. A statistical comparison between all datasets of two individual FRAP/FLIP experiments are depicted in each plot and are illustrated as a single red circle which marks the p-value (y axis) for all intensity values measured for each time point (x axis). The top and bottom dotted lines indicate where p-value equals 0.05. (b) Statistically significant difference between the FRAP dynamics of YFP-β-catenin in the nucleus under Wnt3a treatment versus overexpression of YFP-β-catenin that enters the nucleus without signal, and (c) between the FRAP and (d) FLIP import and export dynamics (refers to Figure 2—figure supplement 1). (e) No statistically significant difference between YFP-β-catenin at the cell membrane between mock-treated and Wnt3a-treated cells (refers to Figure 5—figure supplement 1). (f) No statistically significant difference between YFP-β-catenin at the cell membrane between mock-treated and LiCl-treated cells (refers to Figure 5—figure supplement 1). DOI: http://dx.doi.org/10.7554/eLife.16748.034 [file elife-16748-supp1.jpg]
